# Anterograde Axonal Transport in Neuronal Homeostasis and Disease

**DOI:** 10.3389/fnmol.2020.556175

**Published:** 2020-09-18

**Authors:** Laurent Guillaud, Sara Emad El-Agamy, Miki Otsuki, Marco Terenzio

**Affiliations:** Molecular Neuroscience Unit, Okinawa Institute of Science and Technology Graduate University, Okinawa, Japan

**Keywords:** kinesin, intracellular transport, axon growth, synaptogenesis, neurodegeneration, local translation, liquid phase separation

## Abstract

Neurons are highly polarized cells with an elongated axon that extends far away from the cell body. To maintain their homeostasis, neurons rely extensively on axonal transport of membranous organelles and other molecular complexes. Axonal transport allows for spatio-temporal activation and modulation of numerous molecular cascades, thus playing a central role in the establishment of neuronal polarity, axonal growth and stabilization, and synapses formation. Anterograde and retrograde axonal transport are supported by various molecular motors, such as kinesins and dynein, and a complex microtubule network. In this review article, we will primarily discuss the molecular mechanisms underlying anterograde axonal transport and its role in neuronal development and maturation, including the establishment of functional synaptic connections. We will then provide an overview of the molecular and cellular perturbations that affect axonal transport and are often associated with axonal degeneration. Lastly, we will relate our current understanding of the role of axonal trafficking concerning anterograde trafficking of mRNA and its involvement in the maintenance of the axonal compartment and disease.

## Introduction

**Figure 1:**
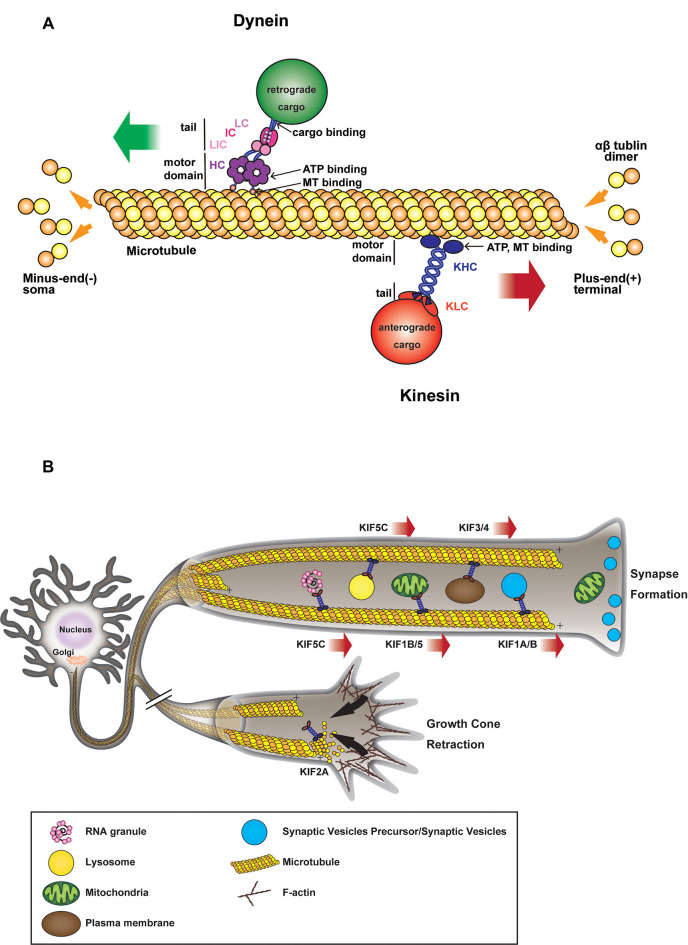
**(A)** Microtubule (MT)-based transport machinery. Schematic representation showing how different molecular motors move along MTs toward MT plus-end (kinesins) or MT minus-end (dynein). Kinesins and dyneins motor domain bind to MT through their globular head domains which hydrolyze ATP during movement. Anterograde or retrograde cargoes bind to the tail domain of the motor either directly or through light/intermediate chains or adaptors. **(B)** Kinesin-mediated anterograde transport during axon elongation and synaptogenesis. Anterograde microtubule-dependent movements of membranous organelles and RNA granules are supported by various plus-end-directed kinesin motors. Organelles such as mitochondria, vesicles, RNA are transported from the soma toward axon tip during axonal growth and synapse formation. In the absence of motor activity, some kinesins also contribute to MT depolymerization during growth cone retraction.

From the discovery of kinesin-1 (Vale et al., 1985) and cytoplasmic dynein (Paschal et al., 1987) in the late 20th century and their initial characterization as anterograde and retrograde motors, respectively (Hirokawa et al., 1990, 1991), substantial effort has been made to decipher their role in neuronal development, connectivity, and synaptogenesis. Since neurons are highly polarized cells with a heavily arborized dendritic network and an elongated axon that can extend over a meter away from their soma, they rely extensively on efficient intracellular transport for the targeting and sorting of proteins and organelles from the soma to their neurite network, where the transfer of information between presynaptic neurons and postsynaptic cells occurs (Südhof, 2018). The somatodendritic and axonal domains have distinct traffic properties and show selectivity towards specific populations of carrier vesicles (Farías et al., 2015). Indeed most somatodendritic vesicles fail to enter the axonal compartment at the level of the axon initial segment (AIS), a highly ordered specialized region of the proximal axon, which acts as a barrier to the diffusion of proteins and lipids between the two compartments (Farías et al., 2015). Long-range trafficking is largely performed by several motor proteins of the kinesin superfamily and cytoplasmic dynein (Hirokawa and Tanaka, 2015; Reck-Peterson et al., 2018). Kinesins mostly deliver their cargoes toward the periphery, while dynein moves in the opposite direction toward the center of the cell (Figure 1A).

Intracellular transport is a fundamental mechanism underlying a variety of neuronal processes, including the establishment of cell polarity, axon growth or regeneration, synaptogenesis, and synaptic transmission and plasticity. Thus, axonal transport has been extensively studied in the past decades. Although the biochemical mechanisms of molecular motors-based transport are well understood, many of the regulatory pathways remain poorly understood, particularly in connection with pathology. It is not surprising to observe that axonal transport perturbations are often associated with severe neurodegenerative pathologies, though whether they are the direct cause or the result of these pathologies remains an open question. Indeed, the nervous system can be affected by a variety of adult-onset neurodegenerative diseases, which are characterized by early synaptic deficit and neurite dysfunction, a phenomenon referred to as “dying back” (Brady and Morfini, 2017). Thus, axonal homeostasis is often affected well before degenerative symptoms can manifest themselves at the level of the neuronal soma. Several pieces of evidence have shown a correlation between mutation of components of the transport machinery (microtubule, molecular motors, and molecular adaptors) and the genesis of neurodevelopmental and neurodegenerative diseases (Maday et al., 2014; Beijer et al., 2019; Sleigh et al., 2019). Also, impairment of axonal transport has been reported in a multitude of neurological disorders that are not directly linked to mutations of proteins belonging to the transport machinery (Sleigh et al., 2019).

Some cargoes are transported along axons anterogradely, some retrogradely and some bidirectionally. Synaptic vesicles, neurofilaments (NFs), and cytosolic proteins are examples of cargoes transported in anterograde fashion while signaling endosomes, autophagosomes, and injury signals are transported retrogradely (Olenick and Holzbaur, 2019). Mitochondria, certain endosomal populations, lysosomes, and mRNAs are transported in a bi-directional manner (Olenick and Holzbaur, 2019). Adaptor proteins selectively recruit molecular motors to specific cargoes targeting them to different transport pathways, which are often interdependent if not convergent (Jean and Kiger, 2012). Interestingly, the aforementioned routes of cargo transport in axons are also taken advantage of by external pathogens such as viruses (Taylor and Enquist, 2015). Even though the two routes are often interdependent as previously mentioned, we will concentrate on the mechanisms of anterograde axonal transport of membrane-bound and membrane-less organelles in neuronal physiology, focusing on several key aspects of axonal growth and synaptogenesis, and other cellular mechanisms such as local mRNA translation and liquid phase separation (LPS) that are likely to be fundamental actors in the regulation of axonal homeostasis and functions. We will also address the links between axonal transport dysfunctions and neurodegeneration, focusing on few neurodegenerative diseases as an example of how defects in anterograde axonal transport can result in neurodegeneration. Though outside of the scope of this review, an extensive wealth of evidence links neurodegeneration and retrograde axonal transport. For extensive coverage of these pathologies and their link to intracellular transport please refer to these comprehensive reviews (Schiavo et al., 2013; De Vos and Hafezparast, 2017; Beijer et al., 2019). We will also briefly discuss the contribution of the cytoskeleton as a necessary platform to facilitate long-range trafficking of mitochondria, which, while moving bidirectionally, need to be addressed as they represent the main source of energy for intracellular transport.

## Cytoskeletal Elements of Axonal Transport

Due to their extremely polarized morphology and their status of postmitotic cells, neurons need to maintain a solid structural cytoskeleton, which is composed of microtubules (MTs), intermediate filaments, and actin filaments. This structure is fundamental to neuronal function and its disruption is associated with neurodegeneration (Beijer et al., 2019).

Active axonal transport of proteins and membranous organelles takes place along MTs (Weisenberg, 1972; Desai and Mitchison, 1997), upon which molecular motors of the kinesin superfamily (Vale et al., 1985; Hirokawa et al., 1989; Lawrence et al., 2004), and cytoplasmic dynein (Paschal and Vallee, 1987; Reck-Peterson et al., 2018) are loaded (Figure 1A). Axonal MTs are longitudinally aligned with their growing plus-end directed towards the axon tip; a large number of kinesins are moving from MT minus to plus-end in a processive manner, while dynein goes in the opposite direction (Howard et al., 1989; Wang et al., 2015). In addition to MTs, NFs are the most abundant cytoskeletal component in axons and control axonal diameter (Grant and Pant, 2000). NFs are formed by neurofilament light (NF-L), medium (NF-M), and heavy (NF-H) chains, apart from the peripheral nervous system, where they contain peripherin as well (Grant and Pant, 2000). While kinesins and dynein are MT associated motors, a third family of molecular motors, myosins, is reliant on actin filaments (Xiao et al., 2016; Beijer et al., 2019). Interestingly, Myosin Va can couple MT and Actin filament-based transport *via* its interaction with Kinesin heavy chain and NF-L, thus helping to regulate the cargo distribution across the cytoskeleton (Cao et al., 2004; Rao et al., 2011).

### Neuropathologies Related to Cytoskeletal Defects

In light of their essential structural function in axons, NFs are critical for axonal transport. NF-L in particular has been shown to regulate NF integrity and their axonal transport (Yates et al., 2009). Not surprisingly, alteration of cytoskeletal elements has been described in several neurodegenerative diseases, where either cytoskeletal proteins or their adaptor/regulators are mutated (Beijer et al., 2019). Perhaps one of the best examples of such pathologies is the Charcot-Marie-Tooth disease (CMT), which is the most common hereditary neuropathy, characterized by distal muscular atrophy and sensory loss (Züchner and Vance, 2006). CMT subtype E (CMT2E) is associated with mutations affecting the integrity of the neuronal cytoskeleton, where mutant NF-L disrupts neurofilament assembly and axonal transport (Jordanova et al., 2003; Lancaster et al., 2018), which in turn perturbs mitochondrial distribution, determining their accumulation within cell bodies and proximal axons (Brownlees et al., 2002). A recessive nonsense mutation was identified in an early-onset CMT patient, which causes a nearly total loss of NF-L mRNA and the subsequent depletion of NF-L protein in patient’s cultured neurons (Sainio et al., 2018). Mutations of different functional NF-L domains were also shown to have different effects on filament assembly, with the *Q333P* mutation leading to reduced NF dimerization (Gentil et al., 2013), while the *P8L* mutation of the head domain affects NF-L phosphorylation, resulting in the destabilization of NF complexes (Brownlees et al., 2002).

NF-H mutations have also been implicated in CMT. A frameshift variant of NF-H leading to the translation of the 3′UTR has been described in families affected by CMT (Rebelo et al., 2016) and shown to result in prominent intracellular protein aggregation, affecting motor neuron viability (Rebelo et al., 2016). These aggregates are recognized by the autophagic pathway, triggering caspase 3 activation, and apoptosis (Jacquier et al., 2017).

While CMT has been associated with direct mutations of cytoskeletal proteins, disruption of MTs can also occur indirectly as a consequence of the mutation of partner proteins that act as MT adaptors and/or interactors. Indeed, mutations of the small heat shock protein HSPB1 and HSPB8 cause distal hereditary motor neuropathy (dHMN) and CMT, and are associated with cytoskeletal abnormality (d’Ydewalle et al., 2011; Irobi et al., 2012; Bouhy et al., 2018). *S135F* and *P182L* mutations of HSPB1 were shown to decrease acetylated α-tubulin abundance, severely affecting axonal transport (d’Ydewalle et al., 2011). Furthermore, HSPB1-*P182L* mutation affects the assembly and transport of NFs, leading to the formation of intracellular aggregates, which include NF-M (Ackerley et al., 2006).

Though the list of neuronal pathologies displaying cytoskeletal defects is constantly growing, we would like to discuss briefly two additional diseases, as an example of pathologies where alteration of cytoskeletal elements is a hallmark of the disease.

Hereditary spastic paraplegia (HSP) is a pathology that leads to axonal degeneration in the corticospinal tracts and, to a lesser extent, in the dorsal column fibers (Shribman et al., 2019). HSP displays perhaps one of the strongest examples of the correlation between defective axonal transport and neurodegeneration (Dion et al., 2009) since most of the genes implicated in HSP encode for proteins that are engaged in intracellular trafficking. The most prevalent form of autosomal dominant HSP stems from point mutation or deletion in the SPG4 gene encoding spastin, a protein involved in MT severing (Roll-Mecak and Vale, 2008). Spastin deletion in mice resulted in defective axonal trafficking, manifested as the accumulation of organelles and NF into focal swellings found exclusively in axonal regions that exhibited fast transition between MT stabilization states (Tarrade et al., 2006). Furthermore, spastin mutants fail to sever MTs, leading to the mislocalization of intracellular organelles (McDermott et al., 2003). A spastin isoform has also been shown to significantly impair fast axonal transport (Solowska et al., 2008) *via* the activation of kinases and phosphatases that play a major role in regulating motor proteins binding to MT and cargoes (Leo et al., 2017). Alteration of MT bundling could also contribute to the disease since spastin was described to be able to bundle MTs *in vitro* (Salinas et al., 2007).

Interestingly, NFs are used as a clinical biomarker in a sporadic and clinical trial for several neurodegenerative diseases, including ALS (Loeffler et al., 2020). Indeed, accumulation of intermediate filament proteins including peripherin is a common pathological feature in both sporadic and familial ALS (Figlewicz et al., 1994; Tomkins et al., 1998; Al-Chalabi et al., 1999; Gros-Louis et al., 2004). NF-H side arm phosphorylation has been reported to slow down the axonal transport of NF by increasing its pausing (Ackerley et al., 2003). Alteration in the stoichiometry of NF subunits has been linked to ALS, while NF side arm phosphorylation is induced by excitotoxic glutamate-mediated activation of JNK, p38 and CDK-p25 kinase (Bajaj and Miller, 1997; Ackerley et al., 2000, 2004). Overexpression of NF-H, NF-L, or peripherin in mice recapitulated the disease pathological features (Collard et al., 1995; Millecamps et al., 2006). Also, TAR DNA-binding protein 43 (TDP-43), one of the key proteins identified in ALS patients neuronal inclusions, can interact with the neuronal cytoskeleton (reviewed in Oberstadt et al., 2018; Hergesheimer et al., 2019), has been shown to alter the stability of NF-L mRNA when mutated (Volkening et al., 2009; Prasad et al., 2019) and impair trafficking and anterograde transport of messenger ribonucleoprotein (mRNP) granules (Alami et al., 2014). Furthermore, loss of function mutations in tubulin alpha 4A protein (TUBA4A) that disrupt MT stability and diminish their repolymerization have been documented in familial ALS cases (Smith et al., 2014), though their impact on axonal trafficking has not been fully elucidated yet. However, since MT stability is central to axonal trafficking, it is likely to be detrimental.

## Molecular Drivers of Anterograde Axonal Transport and Their Role in Neurodegenerative Diseases

### The Kinesin Family of Molecular Motors

A total of 45 genes organized in 15 families are associated with kinesins (also called KIFs) in the human genome (Miki et al., 2001; Lawrence et al., 2004; Hirokawa and Tanaka, 2015; Nabb et al., 2020; Table 1). Kinesin-1, kinesin-2, kinesin-3 and to a lesser extent kinesin-4 subfamily members are implicated in both fast (50–400 mm/day) and slow (less than 8 mm/day) axonal transport (Maday et al., 2014). Fast axonal transport traffics membranous organelles, proteins, and mRNA granules, while slow axonal transport moves MT/NF fragments or other cytosolic proteins necessary for the establishment of neuronal polarity, axon growth and synapse formation (Hirokawa and Tanaka, 2015; Nabb et al., 2020). Plasma membrane proteins generally originated in the rough endoplasmic reticulum at the level of the neuronal soma, must also be delivered peripherally by specialized transport vesicles, and be sorted separately, depending on their axonal or dendritic localization (Bentley and Banker, 2016; Nabb et al., 2020). Kinesin complexes are composed of a globular motor domain, which binds and moves along the MT lattice upon ATP hydrolysis (Hua et al., 1997; Schnitzer and Block, 1997; Kon et al., 2005; Wang et al., 2015), and a tail domain that contributes to the motor auto-inhibition mechanism and the recruitment of various cargoes either directly or through interaction with intermediate scaffolding complexes (Hirokawa et al., 2010). It has been reported that a single cargo could be associated with several motors proteins and the resulting force produced by the ratio between plus-end and minus-end directed motors might determine the final directionality of the movement (Kural et al., 2005; Hendricks et al., 2010), however, only a few cargoes were addressed in this work and it remains unclear whether these findings extend to other cargoes as well. The binding of the cargoes to the motor complex *via* kinesin light chains in the soma, and their release at their final destination, often depends on phosphorylation/dephosphorylation of the motor (Horiuchi et al., 2007; Guillaud et al., 2008; Verhey and Hammond, 2009).

**Table 1:** Kinesin superfamily classification.

Kinesin family	KIFs	Reported axonal localization	References
Kinesin-1	KIF5A, KIF5B, KIF5C	KIF5A, KIF5B, KIF5C	(Xia et al., 2003; Colin et al., 2008; Ma et al., 2009; Nakajima et al., 2012; Su et al., 2013; Campbell et al., 2014; Xiao et al., 2016)
Kinesin-2	KIF3A, KIF3B, KIF3C, KIF17	KIF3A, KIF3B, KIF3C	Takeda et al. (2000) and Nishimura T. et al. (2004)
Kinesin-3	KIF1A, KIF1B, KIF1C, KIF13A, KIF13B, KIF14, KIF16A, KIF16B	KIF1A, KIF1B, KIF3B	Okada et al. (1995), Yonekawa et al. (1998), Miller et al. (2005), Horiguchi et al. (2006), Niwa et al. (2008), Yoshimura et al. (2010), Lo et al. (2011), Kondo et al. (2012), Bharat et al. (2017), Zhang et al. (2017) and Stucchi et al. (2018)
Kinesin-4	KIF4A, KIF4B, KIF7, KIF21A, KIF21B, KIF27	KIF4A, KIF21A, KIF21B	Sekine et al. (1994), Midorikawa et al. (2006), Bisbal et al. (2009), Lee et al. (2012), van der Vaart et al. (2013), Heintz et al. (2014) and Swarnkar et al. (2018)
Kinesin-5	KIF11	KIF11	Swarnkar et al. (2018)
Kinesin-6	KIF20A, KIF20B, KIF23	KIF20B, KIF23	Lin et al. (2012), Sapir et al. (2013) and McNeely et al. (2017)
Kinesin-7	KIF10		
Kinesin-8	KIF18A, KIF18B, KIF19		
Kinesin-9	KIF6, KIF9		
Kinesin-10	KIF22	KIF22	Park et al. (2016)
Kinesin-11	KIF26A, KIF26B	KIF26A	Zhou et al. (2009) and Wang et al. (2018)
Kinesin-12	KIF12, KIF15		
Kinesin-13	KIF2A, KIF2B, KIF2C, KIF24	KIF2A	Morfini et al. (1997), Homma et al. (2003, 2018) and Pfenninger et al. (2003)
Kinesin-14	KIF25, KIFC1, KIFC2, KIFC3	KIFC1	Muralidharan and Baas (2019)

*The members of each family are listed, including KIFs that have been documented in axons (separate columns). References are given for the axonal KIFs*.

While the motor domain is highly conserved and well-characterized in its structure and function (Sweeney and Holzbaur, 2018), the tail domain is more variable and less understood (Nabb et al., 2020). Most work regarding the tail domain characterization has been the focus on kinesin-1 (KIF5A/B/C) and kinesin-3 (KIF1A) family members, which are most studied motors responsible for anterograde transport in the axon, and for which a large number of adaptor proteins mediating their binding to a different population of vesicles has been identified (Verhey et al., 2001; Setou et al., 2002; Wang and Schwarz, 2009; Fu and Holzbaur, 2014). Our knowledge of adaptor proteins for other kinesin families is less defined and the complexity of these interactions is enhanced by the number of vesicle populations in neurons and the need for fine sorting compounded by the extreme neuronal morphology. Indeed, selective anterograde transport in axons and dendrites is essential for the maintenance of neuronal function and polarity, as proteins and vesicles move in one of these compartments and are excluded from the other (Nabb et al., 2020). We will address some of the adaptor proteins involved in this sorting in the following sections, but a more extensive and detailed coverage can be found in this review (Nabb et al., 2020).

The regulation of transport initiation is a critical aspect of kinesin’s ability to mediate axonal transport. Indeed, free cytosolic kinesin-1 and 3 are blocked in an autoinhibited state and can only bind to MTs after a conformational change made possible by their interaction with their cargo (Guedes-Dias and Holzbaur, 2019). Said binding depends on electrostatic interactions between kinesin and tubulin (Woehlke et al., 1997), and the interaction between motor and MTs seems to be stronger for kinesin 3 compared to kinesin-1 (Okada and Hirokawa, 2000; Atherton et al., 2014; Soppina and Verhey, 2014; Lessard et al., 2019). The nucleotide state of MTs can also influence the binding of kinesin-3, which displays higher affinity for GTP-like MTs (Guedes-Dias et al., 2019), while kinesin-1 preferences are still unclear (Nakata et al., 2011; Li et al., 2017; Guedes-Dias et al., 2019). A well-known example of MAPs, which has been reported to inhibit the binding and motility of kinesin-1 is Tau (Dixit et al., 2008; Kellogg et al., 2018; Monroy et al., 2018). Interestingly, Tau mutations account for approximately 50% of cases of Frontotemporal Dementia and Parkinsonism linked to chromosome 17 (FTDP-17), which is characterized by progressive dementia with gradual functional decline (Siuda et al., 2014; Ikeda et al., 2019). However, a large percentage of familiar FTDP-17 are also associated with concurrent mutation of the progranulin (GRN) gene linked to a similar region on chromosome 17 (Forrest et al., 2018). MAP7 on the other hand facilitates the binding of kinesin-1 to MTs *via* its interaction with the stalk domain (Monroy et al., 2018; Hooikaas et al., 2019). Said interaction was recently shown to be important for axonal sorting of cargoes, as MAP7D2 isoform preferentially localizes to MTs in the proximal axon region, where it recruits kinesin-1 (Pan et al., 2019).

### Kinesin-Based Transport Role in Axonal Growth, Brain Wiring, and Neuronal Development

After the establishment of neuronal polarity, axonal elongation is sustained by the addition of membranes to neurite growing tips (Figure 1B). Indeed, plasma membrane precursors and vesicles transported by kinesin-driven axonal anterograde transport from the soma toward the growth cone are crucial to axonal development and wiring (Guedes-Dias and Holzbaur, 2019). KIF13B, for example, anterogradely transports PIP3-containing vesicle, regulating the establishment of neuronal polarity (Horiguchi et al., 2006). Knockdown of KIF13B in hippocampal neurons results in an “axonless” phenotype and Par1b/MARK2-mediated phosphorylation of KIF13B was shown to mediate axon formation (Yoshimura et al., 2010). In PC12 cells, KIF2 deletion inhibits anterograde transport of membranous vesicles and associated receptors, negatively impacting neurite outgrowth (Morfini et al., 1997). KIF2-dependent translocation of IGF-1 receptor stimulates membrane expansion and axonal assembly at growth cone *via* exocytosis of plasmalemmal precursor vesicles in hippocampal neurons (Pfenninger et al., 2003). KIF3 and KIF4 have also been shown to transport membranous organelles through the interaction with fodrin (Takeda et al., 2000) and an unidentified binding protein (Sekine et al., 1994) respectively. KIF3A mediates the transport of PAR-3 to the distal tip of axon in hippocampal neurons, where disruption of PAR-3-KIF3A binding significantly impairs the establishment of neuronal polarity (Nishimura T. et al., 2004). Recently, anterograde axonal transport of lysosome-related organelles is critical for presynaptic biogenesis (Vukoja et al., 2018). Indeed, loss of the kinesin adaptor Arl8 was found to result in an impaired delivery of essential components to the presynaptic site, leading to defects in neurotransmission (Vukoja et al., 2018).

Also, in order to deliver additional plasma membrane to axon tips, axonal transport traffics cytoskeletal components, and mitochondria, providing the structural framework and energy required to support axonal growth (Maday et al., 2014). In *Zebrafish*, KIF5A transports mitochondria into sensory axons through its C-terminal interaction with the adaptors Trak1 and Miro1/2 (Campbell et al., 2014). Mutation in KIF5A significantly reduces the proportion and speed of anterogradely moving mitochondria, resulting in a deficit in axonal mitochondria, which promotes axonal degeneration. In addition to fast axonal transport of mitochondria, KIF5A is also involved in slow axonal transports of NFs. Indeed, NF-H, NF-M, and NF-L accumulate in the soma of peripheral sensory neurons in KIF5A inducible knock-out mice. Such somatic accumulation of neurofilament proteins results in axonal reductions, loss of large-caliber axons, and degeneration (Xia et al., 2003; Xiao et al., 2016). Interestingly, KIF5A participates in both fast (mitochondria) and slow (NFs) anterograde axonal transport, simultaneously contributing to the delivery of energy and the structural scaffolds necessary for the elongation and maintenance of axon growth.

Another kinesin, KIF4A, carries integrin β1 into immature axons. Indeed, It was shown that depletion of KIF4A by shRNA negatively impacts the level of integrin β1 in developing axons and reduces axon elongation in embryonic neurons (Heintz et al., 2014), highlighting the essential role of integrin transport in axonal elongation and initial wiring between immature neurons. It has been previously reported that KIF4A also acts as a regulator of neuronal survival through its interaction and suppression of PARP1 activity in the nucleus; indeed, membrane depolarization induces CaMKII-Ca^2+^ phosphorylation of PARP1, determining its activation after dissociation from KIF4A (Midorikawa et al., 2006). Activation of PARP1 protects mature neurons from apoptosis and allows KIF4A to translocate into the cytoplasm to participate in active transport (Midorikawa et al., 2006). Taken together these observations support a dual function of KIF4A during neuronal development, with KIF4A promoting axonal elongation and connectivity in immature neurons, while protecting mature neurons from apoptosis, thus stabilizing a functional neuronal network. KIF4 was shown to transport anterogradely the P0 protein component of ribosomes along axons (Bisbal et al., 2009). Knockdown of KIF4 in dorsal root ganglion neurons leads to the accumulation of ribosomes in the soma and their disappearance from axons (Bisbal et al., 2009), negatively impacting axonal local protein translation.

Various cellular processes promote growth cone retraction and axonal degeneration of collaterals and branches that failed to establish functional contacts. Kinesin-13 family members, including KIF2A and KIF2C, are important for the homeostatic regulation of neuronal connectivity and brain wiring. KIF2A particularly, while in absence of detectable motor activity, acts as MTs depolymerizer in growth cones to suppress axon collaterals (Homma et al., 2003). Indeed, it has been shown that conditional knock-out of KIF2A promotes mossy fiber sprouting and dendro-axonal conversion of dentate gyrus (DG) cells with aberrant over-extended dendrites gradually acquiring axonal properties in the DG (Homma et al., 2018). Thus, while lacking anterograde motor activity, KIF2A appears to be an essential regulator of neuronal connectivity and the establishment of precise postnatal hippocampal wiring, by determining the pruning of growth cones failing to connect to their postsynaptic target (Figure 1B).

### Axonal Trafficking During Synaptogenesis and Synaptic Transmission

In addition to the establishment and stabilization of neuronal connections, the formation and maintenance of functional synapses are also largely dependent on axonal transport mechanisms. Indeed, synaptic vesicle precursors (SVPs) are known to be transported anterogradely by members of kinesin-3 family, such as KIF1A and KIF1B (Okada et al., 1995). In KIF1A knock-out mice, neurons accumulate SVPs in the soma and fail to establish normal synaptic connections (Yonekawa et al., 1998), while overexpressing KIF1A promotes the formation of presynaptic terminals (Kondo et al., 2012). SVPs are transported by KIF1A and KIF1B *via* either liprin-α or DENN/MADD scaffolding complexes (Miller et al., 2005; Niwa et al., 2008). After the delivery to a presynaptic bouton, SVPs can be recycled directly in the terminal (Miller et al., 2005). KIF1A is also believed to contribute to the active transport of synaptic vesicles between neighboring presynaptic release sites, a pool of vesicles referred to as synaptic vesicle “super pool” (Staras et al., 2010). In cultured giant presynaptic terminals, an axosomatic relay synapse in the auditory brainstem considered one of the largest mammalian excitatory synapses, where MTs depolymerization significantly disrupts the fast-directional transport of the vesicles between neighboring release sites, KIF1A has been found to colocalize with two synaptic vesicles markers, synaptophysin and VGLUT1 (Guillaud et al., 2017). These observations suggest that, in mature synapses, KIF1A-mediated transport plays a significant role in the trafficking and delivery of SVPs and fully functional synaptic vesicles during synaptic transmission (Figure 1B). Indeed, KIF1A homolog Unc-104 is involved in synapse maturation and synaptic transmission (Zhang et al., 2017). KIF1A and KIF1B also contribute to the anterograde transport of dense-core vesicles (DCVs), through interaction with liprin-α (Lo et al., 2011), in a way that is regulated by Ca^2+^ (Stucchi et al., 2018) or through JNK-dependent phosphorylation of synaptotagmin-4 (Bharat et al., 2017). Interestingly, KIF1A associates with DCVs containing Chromogranin-A or BDNF, which move both anterogradely and retrogradely in axons, suggesting that KIF1A might remain attached to DCVs undergoing retrograde transport after the release of BDNF (Stucchi et al., 2018). The anterograde transport from the soma to the synapse of BDNF-containing DCVs is also mediated by KIF5 and its interaction with phosphorylated huntingtin, while their retrograde transport depends on non-phosphorylated huntingtin (Colin et al., 2008). The redundancy of DCVs-transport mechanisms highlights the importance of DCVs targeting and accumulation in the presynaptic compartment and their putative roles in synapse maturation and homeostatic plasticity (Sorra et al., 2006; Tao et al., 2018).

Receptors and voltage-gated channels also need to be efficiently delivered to the synapse to guarantee synaptic transmission. Indeed, conditional KIF5A knock-out mice show behavioral deficits reminiscent of epilepsy, which correlate with a significant reduction in the surface expression of GABA receptors (Nakajima et al., 2012). KIF5A is reported to interact specifically with GABAR-associated protein known to be involved in GABA receptors trafficking, suggesting an important role for KIF5A-mediated transport in inhibitory synaptic transmission. Additionally, KIF5B stalk domain has been shown to directly interact with voltage-gated sodium channel Na1.8 and its overexpression promotes Na1.8 accumulation and neuronal excitability in axons of DRG neurons (Su et al., 2013), suggesting that KIF5B is required for the anterograde transport and function of voltage-gated sodium channels in physiological condition. The correlation between increase in the transport of Na1.8 and KIF5B in pathological conditions, however, needs further investigation (Bao, 2015), and the transport mechanisms of Na1.8 and other sodium channels remain to be fully elucidated. KIF5B-syntabulin-mediated anterograde transport of mitochondria was also shown to be essential for synaptic maturation, basal and sustained neurotransmitter release, and short-term presynaptic plasticity in superior cervical ganglia (SCG) neurons (Ma et al., 2009). Syntabulin is a syntaxin-binding protein that links vesicles to kinesin heavy chain and thus transports syntaxin-containing vesicles into neuronal processes, and its impairment causes a reduction of mitochondria along the axon, correlating with an acceleration of synaptic depression and the slowdown of the recovery rate after synaptic vesicle depletion (Ma et al., 2009).

### Neurodegenerative Diseases Linked to Kinesin Mutations

**Figure 2:**
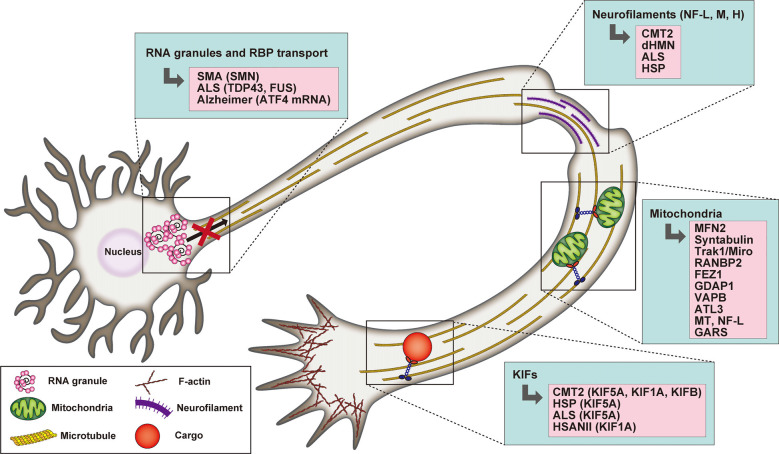
Schematic representation highlighting the association between RNA granule transport, neurofilaments (NFs), mitochondria, and kinesin motors with selected neuronal degenerative diseases. In the case of mitochondria defects, the mutated proteins underlying neurodegeneration are listed.

In support of their fundamental role in driving axonal transport, mutations of kinesin motors are associated with a spectrum of neurodegenerative diseases (Beijer et al., 2019; Figure 2). *De novo* mutations of KIF1A have been found in conjunction with cerebellar atrophy, spastic paraparesis, optic nerve atrophy, peripheral neuropathy, epilepsy and cognitive impairment (Citterio et al., 2015; Esmaeeli Nieh et al., 2015; Lee et al., 2015; Ylikallio et al., 2015; Cheon et al., 2017). Some of these mutations are critical for the structure and function of the motor domain and affect axonal transport (Klebe et al., 2012; Lee et al., 2015; Langlois et al., 2016; Samanta and Gokden, 2019). KIF1A was also found mutated in the hereditary sensory and autonomic neuropathy type II (HSANII), an autosomal-recessive disorder characterized by peripheral nerve degeneration (Rivière et al., 2011). More recently, a missense mutation in KIF1A has been shown to increase excitatory synaptic functions in hippocampal neurons and epileptic seizure-like activity in *Zebrafish*, indicating a direct link between disruption of KIF1A-mediated axonal transport and epileptogenesis (Guo et al., 2020).

KIF5A variants have also been implicated in neurodegenerative diseases such as CMT2, HSP, and ALS (Brenner et al., 2018; Citrigno et al., 2018; Filosto et al., 2018; Nam et al., 2018). Interestingly, the site of the mutation correlates with the clinical phenotype. Indeed, mutations in the motor or neck domain are associated with CMT2 and HSP, while a mutation of KIF5A C-terminus and a mutation that affect splicing are linked to an intermediate slowly progressive form of ALS (Brenner et al., 2018; Citrigno et al., 2018; Filosto et al., 2018; Nam et al., 2018). Several mutations of KIF5A neck and motor domain leading to HSP have been characterized in detail *in vitro* and have been found to exhibit reduced ATPase activity, microtubule affinity and gliding velocity, which affect the processivity and directionality of the motor and can result in reduced cargo flux and consequent deficient synaptic supply (Ebbing et al., 2008; Goizet et al., 2009; Jennings et al., 2017; Dutta et al., 2018).

An autosomal dominant mutation of KIF1Bβ, Q98L, which decreases ATPase activity and motor motility, was initially reported to cause CMT2A in a limited number of pedigrees (Zhao et al., 2001). The lack of confirmation in additional families, however, cast some doubts on the relevance of the mutation (Drew et al., 2015). Recently, a novel KIF1Bβ mutation, Y1087C, was identified in connection with CMT2 (Xu et al., 2018). This mutation was shown to impair the binding between KIF1Bβ and the insulin-like growth factor 1 receptor (IGF1R), affecting IGF1R axonal transport, decreasing its exposure on the neuronal surface and consequently negatively impacting Insulin growth factor 1 (IGF-1) signaling, which is essential for neuronal development and survival (Xu et al., 2018). However, whether this mutation is causative of CMT2 or a polymorphism altering IGF1R trafficking is still an object of debate, since the frequency of the Y1087C mutation is much higher than the total amount of CMT2 cases.

## Refueling Axons and Synapses, Trafficking of Mitochondria and mRNA Granules

### Mitochondrial Trafficking

The homeostatic regulation of axonal growth, neuronal wiring, and synaptic transmission require an extensive amount of energy and rapid protein turnover in the axons, growth cones, and presynaptic terminals, which need to be supported by local production of ATP and proteins along the axon and at the synapse. Axonal transport plays a key role in these phenomena. In addition to the aforementioned syntabulin and Trak1/Miro, RanBP2 (Cho et al., 2007) and FEZ1 (Ikuta et al., 2007) have also been reported to recruit KIF5B and KIF5C to mitochondria and regulate their mobility and trafficking in axons. Interestingly, abnormal co-aggregates of FEZ1 and Kinesin-1 were described in the brains of mouse models of Alzheimer’s disease, suggesting a perturbation of FEZ1-mediated synaptic protein delivery (Butkevich et al., 2016). The existence of several mitochondria adaptor complexes reflects the importance of the axonal transport of mitochondria for the local production of ATP needed to sustain axonal functions (Saxton and Hollenbeck, 2012). Therefore, it is not surprising that, even in the absence of KIF5 mediated transport, a limited fraction of mitochondria is still transported by other kinesins. Indeed, KIF1Bα and KIF1C have been reported to contribute to mitochondria transport through interaction with KBP (Nangaku et al., 1994; Wozniak et al., 2005), as well as KLP6, an uncharacterized kinesin homolog that regulates both mitochondrial morphology and transport (Tanaka et al., 2011).

Transported axonal mitochondria need to remain functional to provide adequate energy support over long distances. Thus, mutations affecting the integrity of mitochondrial morphology and the dynamic balance between their fission and fusion, influence axonal transport (Beijer et al., 2019; Figure 2). Indeed, CMT2A, the most prominent subtype of CMT, is characterized by mutations of mitofusin 2 (MFN2), an outer mitochondrial membrane GTPase that plays a critical role in mitochondrial fusion (Verhoeven et al., 2006). MFN2 has been shown to interact with the Miro/Milton adaptor complex essential for mitochondrial mobilization along MTs. The mutant form disrupts the function of the adaptor complex, thus inducing mitochondrial clustering/aggregation along the axonal length (Baloh et al., 2007; Misko et al., 2010). Interestingly, both mutations in the Miro/Milton complex mediating its interaction with MT and as well as NF-L mutants, indirectly affect mitochondrial transport and localization (Ni et al., 2015). In addition to fusion, dysregulation of mitochondrial fission is also causative of CMT. Recessive mutations of the ganglioside-induced differentiation-associated protein 1 (GDAP1), a mitochondrial factor whose activity is dependent on the fission factors Fis1 and the dynamin-related protein1 (Drp1), determines a reduction in mitochondrial fission activity, while the dominant ones negatively impact mitochondrial fusion (Niemann et al., 2009).

Mitochondrial transport and function are also affected by alteration of the endoplasmic reticulum (ER) and its contacts with mitochondria, where Ca^2+^ exchange between the two organelles occurs. Indeed, disruption of the ER network has been shown to result in axonal degeneration (Yalçın et al., 2017). Mitochondrial Ca^2+^ uptake is required for correct intracellular signaling, homeostasis, and mitochondrial integrity and transport, therefore mutations in Ca^2+^ channels also lead to mitochondrial dysfunction (Kumar et al., 2018). The integral ER membrane protein vesicle-associated membrane protein-associated protein B (VAPB), which is associated to ALS (Nishimura A. L. et al., 2004; Chen et al., 2010), interacts with the outer mitochondrial membrane and its mutation impacts mitochondrial Ca^2+^ uptake and induces the formation of abnormal ER inclusions (De Vos et al., 2012). Interestingly, the ER fusion protein atlastin 3 (ATL3) has been identified in patients with hereditary sensory and autonomic neuropathy (Guelly et al., 2011; Fischer et al., 2014; Kornak et al., 2014). Defects in ATL3 result in an increased number of ER-mitochondria contact sites augmented Ca^2+^ crosstalk between the two organelles and decreased number and motility of axonal mitochondria (Krols et al., 2019).

Mutations in tRNA synthetases, enzymes that attach amino acids to their cognate tRNA molecules in the cytoplasm and mitochondria, affect mitochondrial function and have been associated with a number of human neurodegenerative diseases (Antonellis and Green, 2008; Spaulding et al., 2016). Indeed, Glycyl-tRNA synthetase (GARS) dominant mutations have been described in inherited neuropathies such as CMT2D and dHMN with upper limb predominance (dHMN-V; Xie et al., 2007; Antonellis and Green, 2008). Interestingly, dominant GARS mutations impair neuronal mitochondrial metabolism and cause alterations of VAPB and mitochondrial calcium uptake (Boczonadi et al., 2018). While the disease does not seem to be caused by a loss of the canonical function of these enzymes (Storkebaum et al., 2009; Stum et al., 2011; Ermanoska et al., 2014), mutations of mostly the cytosolic form of tRNA synthetase have been shown to result in toxic gain of function, which impair the signaling output of different families of neurotrophic factor receptors (Stum et al., 2011; He et al., 2015; Sleigh et al., 2017a,b).

### mRNA Axonal Trafficking

We have previously discussed how the correct arrangement of the cytoskeleton and the coordinated action of a cohort of molecular motors are essential for the establishment and maintenance of axonal biology. As axons depend on the delivery of proteins and organelles, fast and local availability of proteins to sustain axonal high turnover rate can also be supported by local translation. While mRNA transport and local protein translation in dendrites have been well documented, the mechanisms of axonal mRNA targeting and translation are still the subject of intense investigation. Indeed, several pieces of evidence have shown that axonally synthesized proteins support axon function, survival, and growth (Sahoo et al., 2018b).

Early observations highlighting how, after detachment from the cell bodies, growth cones were still able to respond to guidance cues in a manner that was dependent on calcium signaling and local protein synthesis, supported the existence of axonal translation (Campbell and Holt, 2001; Ming et al., 2002). The identity and concentration as well as the localization of the cue determine the extent and the nature of the translational response (Brittis et al., 2002; Leung et al., 2006; Manns et al., 2012; Nédelec et al., 2012). Chemotrophic signals, for instance, are known to elicit mRNA transport into axons and growth cones. Indeed, Neurotrophin-3 (NT3) induces targeting and translation of β-actin mRNA into growth cones, which correlates with an increase in growth cone protrusions (Zhang et al., 2001). NGF triggers β-actin mRNA transport into axons (Willis et al., 2005). β-actin is also involved in calcium-mediated growth cone guidance, which is affected by inhibition of β-actin local synthesis or misslocalization of its mRNA (Yao et al., 2006; Welshhans and Bassell, 2011).

RNA-binding proteins (RBP) recognize specific sequence located mostly in the 5′ and 3′ UTR regions of mRNA (emerging evidences implicate the coding region as well), and bind to kinesins or dynein to be transported to axons or dendrites; while the 5′UTR elements are often linked to translation regulation, 3′UTRs regions are essential for targeting to specific subcellular compartments (Hüttelmaier et al., 2005; Chatterjee and Pal, 2009; Merianda et al., 2013; Tushev et al., 2018). The aforementioned β-actin mRNA, for instance, is localized to growth cones by the RBP Zipcode-Binding Protein 1 (ZBP1; Yao et al., 2006). mRNA, RBP and ribosomes are co-transported in large RNA granules, which have been linked to stress granules, where mRNA translation is actively repressed (Kanai et al., 2004; Sahoo et al., 2018a; Pushpalatha and Besse, 2019). These granules display anterograde and retrograde microtubule-based motor movements (Gumy et al., 2014). In addition to KIF5, KIF1Bb might also be involved in mRNA transport, although the mechanism of interaction remains unclear (Lyons et al., 2009).

Axonal injuries are known to trigger local mRNA translation of proteins that will initiate a regenerative transcriptional program in the nucleus through a retrograde signaling cascade originating from the site of injury (Hanz et al., 2003; Perlson et al., 2005; Yudin et al., 2008; Rishal and Fainzilber, 2014; Terenzio et al., 2018). Perturbation of this retrograde mechanisms can cause a delay in axonal regeneration and decrease neuronal survival (Perry et al., 2012; Sahoo et al., 2018b; Terenzio et al., 2018). Nerve injury also induces local translation of mTOR, which in turn controls the axonal synthesis of several retrograde injury signals; thus, disruption of mTOR activity decreases neuronal survival after injury (Terenzio et al., 2018).

Axonal protein synthesis plays also important roles in neurological diseases such as SMA (Spinal Muscular Atrophy), ALS, or Alzheimer’s disease. Indeed, a growing list of mRNAs and RNA binding proteins has been described to be axonally mislocalized in neurodegenerative disease (reviewed in Khalil et al., 2018; Figure 2). For example, loss of SMN significantly alters axonal mRNA levels required for axonal growth and synaptic transmission (Saal et al., 2014; Khalil et al., 2018). Indeed, alterations in the local synthesis of key axonal survival proteins implicated in neurodegenerative diseases have been observed (Kar et al., 2018; Khalil et al., 2018). For instance, expression of the ALS mutants of RNA-binding protein TDP-43 showed decreased mobility of axonal RNPs and reduced axonal transport in motor neurons (Alami et al., 2014), and ALS-causing TDP-43 mutations alter the axonal content of both mRNAs and miRNAs in cultured spinal motor neurons (Rotem et al., 2017). Treatment of hippocampal neurons with amyloid peptide Aβ1–42 promotes axonal translation of Atf4 mRNA and ATF4 retrograde transport leading to neuronal cell death (Baleriola et al., 2014). A recent study showed mRNA translation in axons in connection with late endosomes (Cioni et al., 2019). Interestingly, Rab7a mutants, including those associated with CMT2B, negatively impacted axonal protein synthesis, impaired mitochondrial function, and axonal viability (Cioni et al., 2019). This study highlights the high degree of cross-interaction between different axonal organelles and how these vesicles act as platforms for several signaling pathways as well as different biological cellular functions that have not been associated with intracellular trafficking until recently.

## Conclusions and Perspectives

The combined use of transgenic animal models, primary neuronal cultures, neurons derived from human inducible pluripotent stem cells, in addition to the recent technological advances in proteomics, drug design, and super-resolution microscopy has allowed the in-depth study of the underlying molecular mechanisms behind neurodegenerative diseases (Millecamps et al., 2006; De Vos and Hafezparast, 2017). Many key questions, however, remain open, including the precise molecular identity of the transported vesicles, whether or not it is subjected to change along axons and whether there are any region-specific differences in organelle trafficking within the axonal compartment. Rapid advances in high resolution live imaging *in vitro* and *in vivo* will provide a technological platform to further our knowledge of these phenomena. For example, the trafficking of membrane-less organelles such as stress and/or RNA granules is critical for the maintenance of neuronal homeostasis. The presence of mRNAs granules implies that selected proteins can be locally translated in axons and synapses. Identifying which mRNA can be transported, by which trafficking pathways, and where translation takes place, is, thus, paramount to our understanding of axonal biology. Luckily, several novel proteomic approaches have been designed to identify newly synthesized proteins (Forester et al., 2018; Koppel and Fainzilber, 2018; Terenzio et al., 2018; Holt et al., 2019), together with new imaging tools engineered to visualize localized mRNA and protein translation (Morisaki et al., 2016; Wu et al., 2016). These new technological developments will give us valuable insights into the cooperation between intracellular transport mechanisms and local protein synthesis in both physiological and pathological conditions.

LPS of biomolecules has also recently emerged as a novel fundamental mechanism underlying subcellular organization and regulation (Chen et al., 2020). The formation of highly condensed molecular assemblies, also known as membrane-less organelles or bio-condensates, within aqueous solutions such as the cytoplasm, plays critical roles in the maintenance of neuronal functions and in neurodegeneration (Elbaum-Garfinkle, 2019). The formation of various components of mRNA and/or stress granules that are targeted to and transported in axons have also been shown to be regulated by LPS. Indeed, the Fragile X Mental Retardation Protein (FMRP) undergoes phosphorylation-dependent phase separation with RNA in a synaptic activity-dependent manner to generate membrane-less-RNA-protein transport granules (Tsang et al., 2019). TDP43 low-complexity domain phase-separates to form cytoplasmic stress granules (Babinchak et al., 2019) and the persistence of phase-separated TDP43 independently of stress granules can induce neuronal cell death (Gasset-Rosa et al., 2019). TDP-43-containing axonal mRNA transport granules have also been reported to display liquid-like properties (Gopal et al., 2017). Additionally, synapsin-1 has been demonstrated to phase-separate and promote synaptic vesicles clustering at the synapse regulating the mobility of synaptic vesicles in axon terminals (Milovanovic et al., 2018). Active zone protein RIM-1 has also been shown to undergo a phase transition, which might represent the basic mechanism underlying the organization of release sites at the synapses (Wu et al., 2019). Lastly, LPS of disordered proteins such as Tau in Alzheimer’s disease (Ambadipudi et al., 2017; Wegmann et al., 2018), FUS/TDP43 in ALS (Murakami et al., 2015; Patel et al., 2015; Conicella et al., 2016), huntingtin protein in Huntington’s disease (Peskett et al., 2018) have been recently reported to be critical for their pathological aggregation and toxicity. Similar mechanisms might also be involved in the aggregation of β-amyloid precursor proteins in Parkinson’s disease (Boke et al., 2016; de Gap et al., 2019) and α-synuclein.

Although the contribution of LPS to long-range transport in neurons remains an open question, perturbations in LPS likely affect the formation of phase-separated transport granules (reviewed in Nötzel et al., 2018). A recent study has reported that the long-distance trafficking of mRNA granule/lysosome complex depends on LPS of annexin 11 and that this mechanism is critical for their axonal transport (Liao et al., 2019). Another *in vitro* study also suggested that prolonged LPS of Tau can lead to the formation and aggregation of pathogenic Tau, a form of Tau known to affect axonal transport (Kanaan et al., 2020). Though we have just started to decipher the molecular mechanisms leading to the formation of these bio-condensates, their recruitment onto molecular motors and their targeting to axons and synapses, the discussed pathological aggregation of various neuronal proteins, point to a plausible correlation between perturbations in protein LPS and neurodegeneration. Thus, the integrative study of transport mechanisms, local protein synthesis, and LPSs is critical to reconstructing a comprehensive picture of the multiple cellular and molecular pathways that cooperatively or sequentially take place to efficiently regulate axonal functions.

## Author Contributions

LG, SE-A and MT participated in the design and writing of this review. MO made the figures.

## Conflict of Interest

The authors declare that the research was conducted in the absence of any commercial or financial relationships that could be construed as a potential conflict of interest.
